# L-dopa treatment increases oscillatory power in the motor cortex of Parkinson's disease patients

**DOI:** 10.1016/j.nicl.2020.102255

**Published:** 2020-04-20

**Authors:** Chunyan Cao, Dianyou Li, Shikun Zhan, Chencheng Zhang, Bomin Sun, Vladimir Litvak

**Affiliations:** a Department of Neurosurgery, Affiliated Ruijin Hospital*,* Shanghai JiaoTong University School of Medicine*,* Shanghai, 200025, China; b Wellcome Centre for Human Neuroimaging*,* UCL Queen Square Institute of Neurology, London WC1N 3BG*,* UK

**Keywords:** Dopamine, Cortex, Human, Movement disorders, M1

## Abstract

Parkinson's disease (PD) is a movement disorder caused by dopaminergic neurodegeneration. Levodopa (L-dopa) is an effective medication for alleviating motor symptoms in PD that has been shown previously to reduce subcortical beta (13–30 Hz) oscillations. How L-dopa influences oscillations in the motor cortex is unclear. In this study, 21 PD patients were recorded with magnetoencephalography (MEG) in L-dopa ON and OFF states. Oscillatory components of resting-state power spectra were compared between the two states and the significant effect was localized using beamforming. Unified Parkinson's Disease Rating Scale (UPDRS) III akinesia and rigidity sub-scores for the most affected hemibody were correlated with source power values for the contralateral hemisphere. An L-dopa-induced power increase was found over the central sensors significant in the 18–30 Hz range (*F*_(1,20)_  > 14.8, *P*_FWE corr_ < 0.05, cluster size inference with *P* = 0.001 cluster-forming threshold). Beamforming localization of this effect revealed distinct peaks at the bilateral sensorimotor cortex. A significant correlation between the magnitude of L-dopa induced 18–30 Hz oscillatory motor-cortical power increase and the degree of improvement in contralateral akinesia and rigidity was found (*F*_(2, 19)_ = 4.9, *p*_one-tailed_ = 0.02, *R*^2^ = 0.2). Power in the same range was also inversely correlated with combined akinesia and rigidity scores in the L-dopa OFF state (*F*_(2, 19)_ = 9.2, *p*_two-tailed_ = 0.007, *R*^2^ = 0.33) but not in the L-dopa ON state (*F*_(2, 19)_ = 0.27, *p*_two-tailed_ = 0.6, *R*^2^ = 0.01). These results suggest that the role of motor cortical beta oscillations in PD is distinct from that of subcortical beta.

## Highlights

• L-dopa induces beta power increase over the motor cortex significantly in the 18–30 Hz range.
• Beta power in motor cortex is lower in PD patients with severer akinesia and rigidity impairment.
• The increase of beta power induced by L-dopa is higher in PD patients with better clinical improvement.

## 1 Introduction

According to a well-established model of the motor circuit (Wichmann and Dostrovsky, 2011), neural oscillations from the motor cortex are processed by the basal ganglia (BG), and the output of this processing is transmitted back to the motor cortex via the thalamus, facilitating or inhibiting voluntary motor actions. Dopaminergic innervation of the striatum by neurons from the substantia nigra pars-compacta is essential for healthy information processing in the BG. Parkinson's Disease (PD) is a movement disorder characterized by the loss of nigral dopaminergic neurons. Its specific motor symptoms: tremor, rigidity, akinesia, and postural instability, are caused by the degeneration of these neurons and the subsequent dysfunction of BG motor circuits (McAuley, 2003). Levodopa (L-dopa) substantially alleviates these symptoms. However, as the disease progresses, PD patients become less responsive to L-dopa and develop adverse reactions, leading to a reduced quality of life. Deep Brain Stimulation (DBS) further relieves the motor symptoms in these patients especially tremor, rigidity and akinesia (Benabid et al., 2006). During DBS surgery, recordings of local field potential (LFP) from the BG, particularly in the subthalamic nucleus (STN), have revealed over-synchronized beta oscillations and their amplitude (power) was correlated with patients’ motor impairment. L-dopa and DBS have been shown to suppress this pathological activity in the STN (Giannicola et al., 2010; Kuhn et al., 2009). Compared to the clear consensus regarding the role of subcortical beta synchronization in PD, there is still no general agreement on the role of cortical beta oscillations and how they are affected by L-dopa.

Magnetoencephalography (MEG) noninvasively detects weak magnetic signals induced by electrical activity in the brain, including the cerebral cortex (Cohen, 1968, 1972) and subcortical areas (Boon et al., 2017). The combination of good localization accuracy and high temporal resolution provided by MEG means that oscillatory activity can be studied in great detail (Baillet, 2017). Previous MEG studies showed that the average frequency of cortical oscillations in PD patients decreases over the course of the disease, most prominently in the posterior brain region (Stoffers et al., 2007). In advanced stage of PD, oscillatory slowing was barely influenced by L-dopa administration (Stoffers et al., 2008). Longitudinal investigation of PD patients showed an increase in the power of the “slower” frequencies (theta and alpha 1 band), whilst the power of the “faster” frequencies (beta and gamma) was reduced (Cao et al., 2015).

Regarding specifically the effect of L-dopa on the cortical beta power in PD patients, there have been contradictory findings with some reports suggesting that PD impairment is associated with elevated cortical beta (Hall et al., 2014; Pollok et al., 2012) whilst other studies have reported that beta power is decreased in PD and is elevated by L-dopa administration (Heinrichs-Graham et al., 2014; Melgari et al., 2014) Furthermore, one study has found no significant effect of L-dopa on EEG power (George et al., 2013).

To date, there has been no MEG study looking at the effects of L-dopa on the power of cortical oscillations and correlating these with clinical improvement. The only study following a similar protocol (Melgari et al., 2014) was undertaken using EEG. Here we recorded MEG at rest in a large cohort of PD patients on and off L-dopa who also underwent clinical assessment based on video recordings. We used a recently developed spectral decomposition method making it possible to isolate the oscillatory component of the spectrum from the background noise (Haller et al., 2018). We hypothesize that oscillatory power estimated with this method reflects the clinical state.

## 2 Materials and methods

### 2.1 Subject characteristics

Twenty one PD patients were recruited from Ruijin Hospital Functional Neurosurgery Department (Table 1) and fulfilled the United Kingdom Parkinson's disease Society Brain Bank (UK-PDSBB) clinical diagnostic criteria for probable PD (Gibb, 1988). All patients were free of significant medical, neurologic and psychiatric diseases except for PD. Written consent for participation was provided by all subjects. The study was approved to conform to the Helsinki declaration by the local ethics committee of Affiliated Ruijin Hospital, Shanghai JiaoTong University School of Medicine.

**Table.1:** Characteristics of the enrolled Parkinson's disease patients. The columns are gender, age, disease duration, and the UPDRS III scores of left (l) and right sides (r) of hemibody tremor and the combined rigidity and akinesia at 12 hours’ withdrawal of Parkinsonism medication (OFF) and 1 to 2 h after the administration of L-dopa (ON) states. LED, L-dopa equivalent dose. ys, years

	Age/duration	UPDRSIII	OFF (l/r)	ON (l/r)	OFF (l/r)	ON (l/r)	
#/Sex	(ys)	Total (OFF/ON)	Tremor	Tremor	Rigidity + Akinesia	Rigidity + Akinesia	LED (mg)/Day
1-M	40/5	52/23	0/0	0/0	27.5/30	12.5/13.5	486
2-M	44/5	54/10	0/0	0/0	29/35.5	2/9	800
3-M	60/7	48.5/25.5	0/0	0/0	29/23	15.5/11	600
4-M	65/10	58.5/32	0/2.4	0/0	30.5/32.5	17.5/16	600
5-M	71/9	48/29	0/0	0/0	28.5/27.5	17.5/17.5	750
6-M	34/6	55/21	0/0	0/0	34.5/33	11/13	787.5
7-M	65/12	41.5/20	0/1	0/0	21.5/22	12/11	1075
8-F	66/5	28.5/9	0/0	0/0	19.5/22	6/5	725
9-M	71/13	77.5/39.5	5/5	2/1.5	38/39.5	21/22.5	1262
10-M	54/14	57.5/25.5	2/3.5	0/0	27.5/31.5	13.5/12.5	450
11-F	50/8	38/20.5	2/0	0/0	19/19	8.5/10	550
12-F	68/16	61/32	0/2.5	0/0	37/30.5	19.5/19.5	600
13-M	74/4	40/33	2.5/0	2/0	20.5/20	18/17	475
14-F	61/9	58.5/29.5	5/3.5	0/0	28.5/32.5	16.5/17.5	500
15-M	57/7	61/34.5	0/0	0/0	33.5/36	18/20	300
16-M	55/7	47/31.5	0.5/0.5	0/0	27/24	17/16	525
17-M	67/12	57.5/23.5	0/2.5	0/0	35/34	13.5/13	1200
18-M	62/9	57/15	2.5/0	0/0	30.5/33	5/8	1475
19-M	62/15	48/26.5	0/0	0/0	28/30.5	17/17	650
20-M	67/3	35/8.5	0/3	0/0	19/19	4.5/6.5	400
21-M	62/11	46/16	0/0	0/0	25/30	7/9	700

All patients were recorded with MEG twice (i) after 12 h withdrawal of anti-Parkinson's disease medication (OFF) and (ii) on the same day 1–2 h after the administration of L-dopa (ON). L-dopa was administered immediately after the OFF recording. The L-dopa dose for each patient was 1.5 times the L-dopa equivalent dose (LED) for their normal medication dose.

### 2.2 UPDRS motor scores evaluation

The patients were video recorded in the OFF and ON states before the MEG recording and the recordings were later assessed by a specialist in movement disorders using the Unified Parkinson's Disease Rating Scale (UPDRS III). The general motor impairments were assessed with subscores for rest tremor (sum of UPDRS III sub-item 20 for bilateral hand and foot), rigidity (sum of UPDRS III sub-item 22 for bilateral upper and lower extremities) and akinesia (sum of UPDRS III sub-items 23, 24, 25, 26 for bilateral extremities, and 27, 28, 29, 30, 31 for walking condition). For correlating with neurophysiological measures, hemibody akinesia + rigidity score was used (the sum of sub-items 22, 23, 24, 25, 26 for unilateral extremities and 27, 28, 29, 30, 31).

### 2.3 MEG recordings

The MEG recordings were performed with a 306-channel, whole-head VectorView MEG system (Elekta Oy, Helsinki, Finland) in a magnetically shielded room (Euroshield, Eura, Finland). Head position within the MEG system was determined by digitizing the position of the bilateral pre-auricular and the nasion fiducial points. During recording, the patient rested in supine position with eyes open for 3 min. The raw MEG data were band pass filtered in 0.03–330 Hz range and digitized at a sampling rate of 1000 Hz. The magnetic artifacts and movement artifact were removed by the temporal extension of Signal Space Separation method (tSSS) implemented in the MaxFilter software (Neuromag 3.4, Elekta Oy, Helsinki, Finland). tSSS used a 10 s raw data buffer with a subspace correlation limit of 0.98.

### 2.4 MEG data analysis

#### 2.4.1 Preprocessing

MEG data were analyzed in SPM12 (Litvak et al., 2011b) (https://www.fil.ion.ucl.ac.uk/spm/). Low-frequency drift and line noise were removed by filtering (5th order, Butterworth filter, 1 Hz high-pass, 48–52 Hz bandstop). The data were epoched into non-overlapping 1 s trials and epochs with deflections exceeding 150 fT/mm on any of the planar gradiometers were rejected. The number of epochs included in the analysis was 176 ± 17 for the OFF and 166 ± 22 for the ON state. No gradiometer channels had to be excluded.

#### 2.4.2 Sensor-level statistics

Spectra for each channel were computed from the planar gradiometers in the 0–98 Hz range using a multi-taper method (Thomson, 1982) with spectral resolution of ±2 Hz. The spectra were averaged across trials using robust averaging (Holland and Welsch, 2007; Litvak et al., 2012) and summed for pairs of co-localized orthogonal gradiometers. In order to separate the oscillatory component of the spectrum from the non-oscillatory background component, we used the recently proposed algorithm for Fitting Oscillations and One over F (FOOOF, https://github.com/fooof-tools/fooof (Haller et al., 2018). This algorithm models the log-spectrum as a sum of exponential background component with a possible change in slope (a knee) and a set of Gaussians that fit oscillatory peaks. The algorithm was applied in the 1–90 Hz range after replacing the 45–55 Hz range affected by line noise with a shape-preserving piecewise cubic interpolation. The goodness of fit in the 1–45 Hz range was visually verified for a sample of spectra from different subjects to make sure these settings worked well. Since the number and central frequencies of Gaussians varied between subjects hindering group analysis, we only used FOOOF to subtract the background component. The remainder was used as an estimate of true oscillatory log-power corrected for any physiological or artefactual changes in background noise.

The corrected spectra in the 1–45 Hz range were converted to volumetric images by linearly interpolating the power topographies for each frequency bin into a 2D image (32  ×  32 pixels). The resulting images were stacked to produce a 3D image with two spatial and one frequency dimension. The 3D images were exported to Neuroimaging Informatics Technology Initiative (NIfTI) format, smoothed with a Gaussian kernel with full width at half maximum (FWHM) of 8 mm x 8 mm x 4 Hz and subjected to a paired *t*-test comparing the ON and OFF conditions using a standard SPM approach to sensor-level statistics (Kilner and Friston, 2010). This was our main statistical test for testing for the effect(s) of L-dopa. The results are reported at *p* < 0.05 family-wise error (FWE) corrected at the cluster-level with the cluster-forming threshold of *p* < 0.001.

#### 2.4.3 Source analysis

To identify the sources of significant power differences detected at the sensor level, we used Dynamic Imaging of Coherent Sources (DICS) (Gross et al., 2001) method implemented in the SPM toolbox for data analysis in source space (DAiSS, https://github.com/spm/daiss). The forward computation used the single shell head model (Nolte, 2003) based on SPM template head meshes scaled to fit the individual fiducials and head shape. The source orientation was optimized to yield maximal power. DICS analysis only used planar gradiometers. Note, however, that following tSSS these are informed by both original sensor types. To perform noise normalization at the source level analogous to that done at the sensor level we subtracted from a power image in the range f_1_–f_2_ Hz an image for (f_1_ + 100 Hz) to (f_2_ + 100 Hz). The resulting images were smoothed with a Gaussian kernel (FWHM 8 × 8 × 8 mm) and subjected to a paired *t*-test comparing the ON and OFF conditions. Since this was effectively a post-hoc test informed by the previous sensor-level test we set the statistical threshold at *p* < 0.01 uncorrected which allowed for visualization and separation of the effects in the two hemispheres.

#### 2.4.4 Extraction of source spectral features

We used a Linearly Constrained Minimum Variance (LCMV) beamfomer (Van Veen et al., 1997) to extract virtual electrode time series from the individual peaks of ON-OFF power differences closest to the group peak for each hemisphere. The beamformer orientation was constrained to yield maximum power across both L-dopa conditions. We then computed the spectra for these time series in the range of 1–100 Hz and applied FOOOF correction with the same settings as at the sensor level. The use of LCMV beamformer was necessary to obtain a wideband spectrum used as the input for FOOOF. Separate source extraction from the two hemispheres made it possible to obtain hemisphere specific log-power estimates to correlate with the contralateral hemibody akinesia + rigidity scores. This is important because the degree of impairment in PD is usually asymmetric.

#### 2.4.5 Correlations with clinical scores

The estimates of oscillatory power were computed by averaging the FOOOF corrected source log-spectra in the band where a significant L-dopa effect was found at the sensor level (18–30 Hz, see Results). These estimates were correlated separately with combined akinesia + rigidity scores for the ON and OFF states as well as their difference (Pearson correlation). For each subject we used the most affected hemibody and the contralateral hemisphere for these correlations. The most affected hemibody was defined as the one with the higher akinesia + rigidity score in the OFF state. The results are reported with significance levels set at *p* < 0.05.

## 3 Results

### 3.1 Demographic and clinical characteristics

Twenty-one PD patients (4 women, age: 59.8 ± 10.5 years) were enrolled in the study. The demographic and clinical characteristics of the subjects and their ON and OFF states UPDRS III scores are represented in Table 1. The L-dopa equivalent dose (LED) did not show a significant correlation with the disease duration (Pearson's correlation, *p* = 0.09, one-tailed test).

### 3.2 Effects of dopaminergic challenge on the motor symptoms and cortical oscillations

**Fig. 1:**
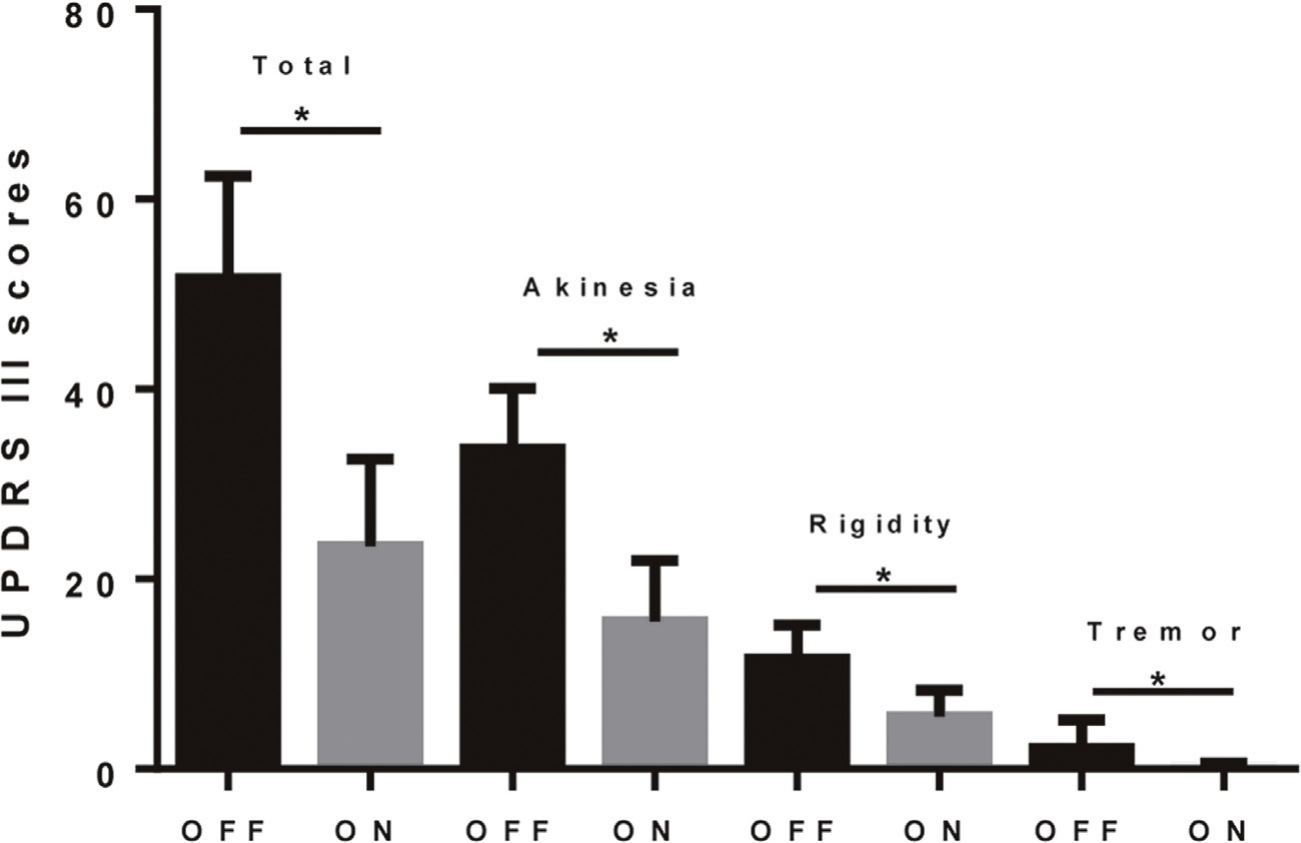
L-dopa significantly alleviated the motor symptoms of PD patients. The asterisks indicate *p* < 0.0001 in a paired *t*-test. The improvement in UPDRS III was 55% in total score, 54% in akinesia, 53% in rigidity and 93% in tremor.

Administration of L-dopa significantly improved the patients’ motor symptoms evaluated by the total UPDRS III score (*p* < 0.0001, paired *t-*test). The improvement in UPDRS III was 55% in total score, 93% in tremor, 53% in rigidity and 54% in akinesia (Fig. 1).

**Fig. 2:**
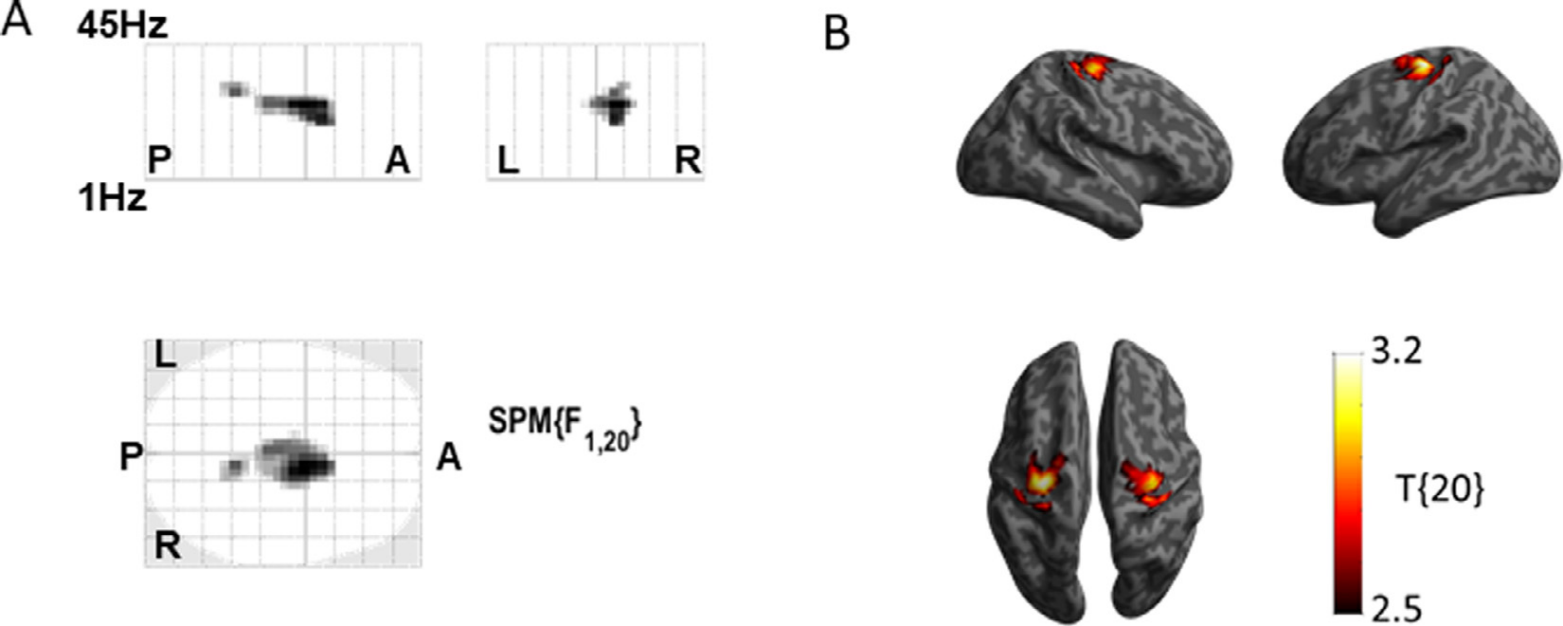
L-dopa increased the beta power in the motor cortex of PD patients. (A). Sensor-level scalp x frequency comparison of MEG power between the ON and OFF states. L-dopa induced significant power increase over the central sensors in the 18–30 Hz range (peak at 24 Hz, *P*_FWE corr_ < 0.05 at the cluster-level with cluster-forming threshold *p* < 0.001). The letters indicate directions to aid interpretation of the map (A-anterior, P-posterior, L-left, R-right) (B). DICS beamforming localization focusing on the significant frequency band revealed distinct power peaks in the bilateral sensorimotor cortex (*P*_uncorr_ < 0.01). The right side showed a peak at Montreal Neurological Institute (MNI) coordinates 36 −22 62 corresponding to Brodmann area (BA) 4. The left side showed a peak at −34 −20 68 corresponding to BA6.

An L-dopa-induced increase in the oscillatory component of cortical log-power was found which was expressed over the central sensors and was significant in the 18–30 Hz range (*F*(_1,20_) > 14.8, *P*_FWEcorr_ < 0.05, cluster size inference with *P* = 0.001 cluster-forming threshold) (Fig. 2A). DICS beamforming localization of this effect revealed distinct peaks at the bilateral motor cortex (*T*(_20_) > 2.53, *P*_ucorr_ < 0.01) (Fig. 2B). The peaks were observed at Montreal Neurological Institute (MNI) coordinates (*x y z*) 36 −22 62 in the right Brodmann area 4 (BA4) and −34 −20 68 in left Brodmann area 6 (BA6) (Fig. 2B).

### 3.3 UPDRS subscores correlations to the cortical oscillations

**Fig. 3:**
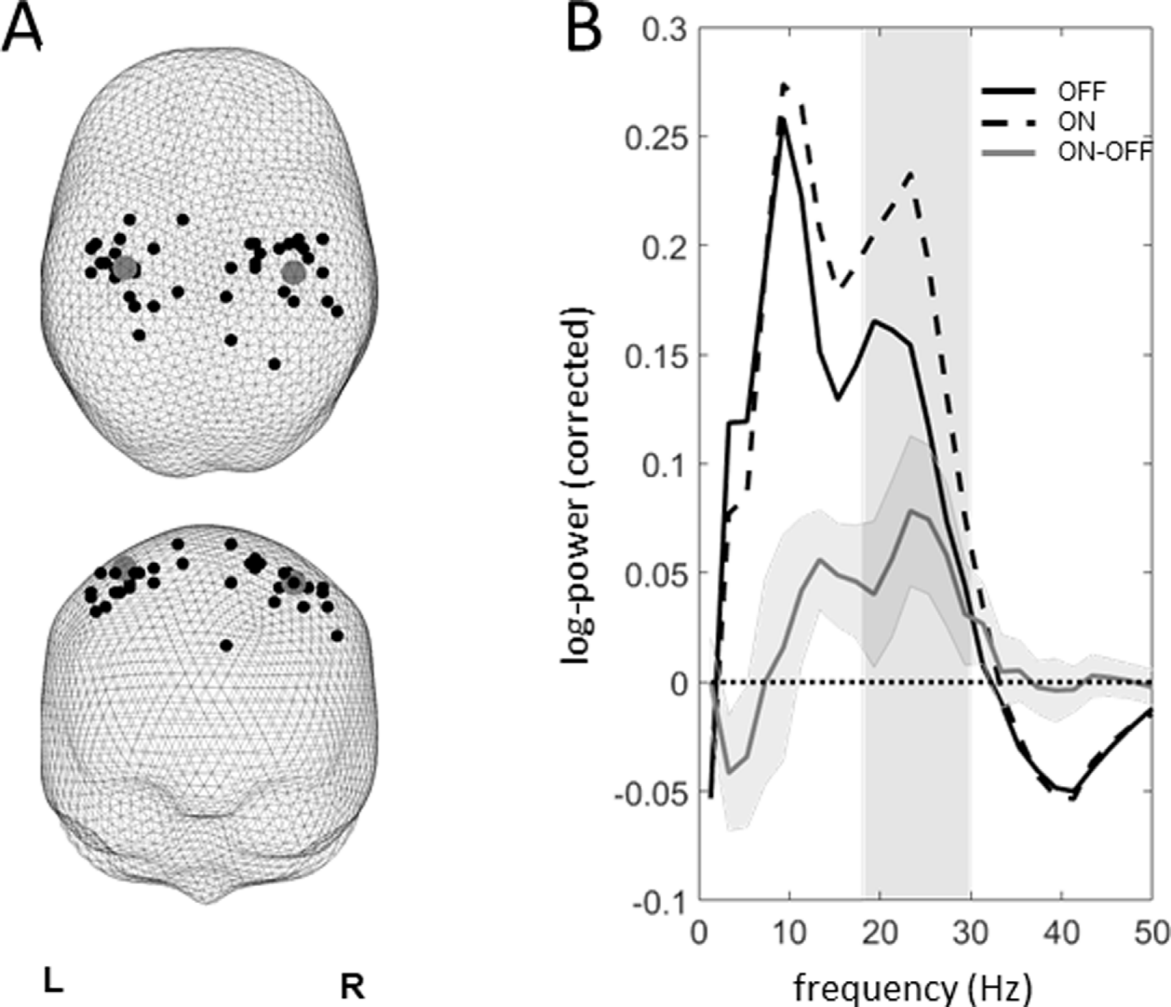
(A). The individual peaks of the ON-OFF difference images used for virtual electrode source extraction. The group peaks are shown with large gray circles. (B). Oscillatory components of the log-power spectra (after subtraction of the non-oscillatory component estimated with the FOOOF algorithm) averaged across hemispheres for the two L-dopa states and their difference. The difference curve is shown with 95% confidence intervals. The shaded area corresponds to the 18–30 Hz range for which a significant effect was found at the sensor level.

To obtain hemisphere-specific power estimates we performed virtual electrode source extraction from the individual hemisphere-specific peaks of the ON-OFF L-dopa difference images closest to the corresponding group peaks (Fig. 3A). Comparing the FOOOF-corrected source spectra for the source data revealed effects consistent with the sensor-level analysis (Fig 3B). The magnitude of the oscillatory component of source log-power spectra was averaged in the range of the significant sensor-level effect (18–30 Hz) and correlated with the corresponding contralateral akinesia + rigidity UPDRS III subscores for the ON and OFF states and their differences. For each subject we used the most affected hemibody and the contralateral hemisphere for these correlations.

**Fig. 4:**
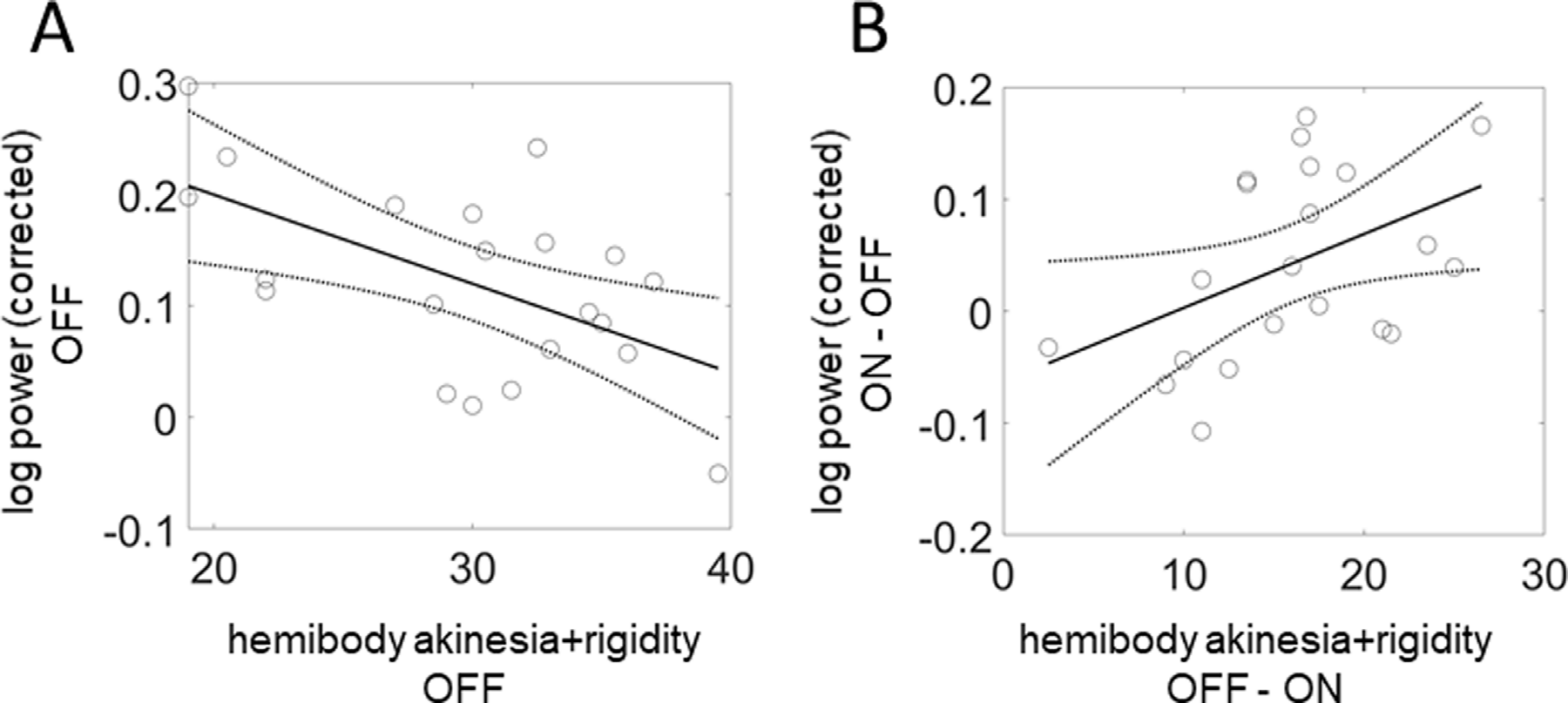
Correlations of the source-level beta power with combined akinesia/rigidity in PD patients. These correlations were computed for the most affected hemibody and the contralateral hemisphere. (A). Significant correlation of 18–30 Hz oscillatory log-power with the contralateral akinesia + rigidity scores in the OFF state (*F*_(2, 19)_ = 9.2, *P*_two-tailed_ = 0.007, *R*^2^ = 0.33). (B). Significant correlation of ON-OFF difference in 18–30 Hz oscillatory log-power with OFF-ON difference in akinesia + rigidity (*F*_(2, 19)_ = 4.9, *P*_one-tailed_ = 0.02, *R*^2^ = 0.2).

A significant negative correlation was found between beta power and akinesia + rigidity in the OFF state (*F*_(2, 19)_ = 9.2, *p*_two-tailed_ = 0.007, *R*^2^ = 0.33, Fig 4A). In addition, a significant positive correlation was found between the degree of increase in beta log-power induced by L-dopa and the contralateral clinical improvement (*F*_(2, 19)_ = 4.9, *p*_one-tailed_ = 0.02, *R*^2^ = 0.2, Fig 4B). The increase in beta log-power was measured by the subtraction ON−OFF, while clinical improvement was measured by the subtraction OFF−ON. No correlation was found between the beta power and akinesia + rigidity in the ON state (*F*_(2, 19)_ = 0.27, *p*_two-tailed_ = 0.6, *R*^2^ = 0.01).

## 4 Discussion

In this study, we found that the L-dopa administration in PD patients bilaterally increases beta (18–30 Hz) power in the motor cortex, peaking at left BA4 and right BA6. L-dopa induced an improvement of 55% in the total UPDRS III score confirming that the PD patients included in the study had good response to L-dopa. The beta power was negatively correlated with combined akinesia + rigidity scores in the OFF state and there was a positive correlation of L-dopa induced power increase with improvement in akinesia + rigidity for the most affected side of the body across patients.

Several studies examined the effect of L-dopa on cortical oscillations in PD using EEG and MEG (for a recent review see Boon et al., 2019 for MEG; Geraedts et al., 2018 for EEG). Melgari et al. (Melgari et al., 2014) found an increase in alpha and beta band EEG power with L-dopa that was correlated with improvement in rigidity and bradykinesia. George et al. (George et al., 2013) reported effects on sensor-level pair-wise coherence where increased coherence in the beta band was observed in the OFF state. Heinrichs-Graham et al. (Heinrichs-Graham et al., 2014) found that beta power was decreased in PD patients (disease duration 5 ± 2.6 (s.d.) years, range 1–9) off medication compared to healthy controls and increased by L-dopa treatment. At the same time, interhemispheric phase synchrony was reduced by L-dopa in line with George et al.’s results. Pollok et al. (Pollok et al., 2012) compared MEG beta power between healthy controls, newly diagnosed medication-naïve PD patients and chronically medicated patients. In both patient groups resting beta power in the motor cortex was significantly higher than in healthy controls and there was no significant difference between the medicated and unmedicated group. Finally, Hall et al. (Hall et al., 2014) showed that in early stage of PD, motor cortical beta power was higher contralateral to the most affected side and this asymmetry was reduced by Zolpidem (a GABA_A_ receptor agonist). A possible unifying explanation for these results is that beta power in cortical motor areas is increased in the early stages of PD relative to controls but decreased in later stages. In these late stage patients, L-dopa acts to increase beta power.

Our findings are consistent with this idea. The disease duration in our cohort was 9 ± 3.7 (s.d.) years. Therefore, these patients can be considered late stage. Like Heinrichs-Graham et al. (Heinrichs-Graham et al., 2014), we found an increase in beta power induced by L-dopa. Additionally, the inverse correlation between beta power and akinesia + rigidity in the OFF state is consistent with the idea that reduced cortical beta is associated with more severe clinical impairment (Melgari et al., 2014). Based on these two findings one would expect that the degree of increase of beta power induced by L-dopa would be positively correlated with akinesia + rigidity improvement, which was indeed the case.

We did not find a significant correlation between beta power and akinesia + rigidity in the ON state. This could be due to ceiling effects. Another possible explanation is that MEG records and clinical evaluation videos were not obtained at the same time point. The motor symptoms varied at different intervals after L-dopa administration. In our study, the video recording and the MEG acquisition started 1 h after the administration of L-dopa. But the interval between the video recording and the MEG acquisition was different in each subject due to clinical and logistic constraints. This variability could act to reduce the correlation between beta power and clinical scores in the ON state. Conversely, in the OFF state which was 12 h after withdrawal of medications, the patients’ condition at video taking and the MEG recording was more consistent.

The limitations of the clinical evaluation by the neurologists are their subjectivity and inaccuracy. In the future, behavioral investigations using wearable devices for clinical evaluation may help overcome these limitations.

Although a subset of our patients (*N* = 12) had tremor, our study design was not optimized to look at its neurophysiological correlates. Patients with severe tremor were not enrolled in this study due to the need to remain motionless in the MEG. Thus both the low patient number and narrow tremor severity range do not allow us to report on correlation of cortical power with tremor although previous studies suggest such correlations could exist (Contarino et al., 2012; Timmermann et al., 2003).

To understand the role of cortical beta oscillations in PD pathology, the effects of L-Dopa should be compared to another common treatment – DBS. Four recent MEG studies have examined the effect of DBS on cortical power. In the beta band, two of the studies reported a beta decrease (Abbasi et al., 2018; Luoma et al., 2018) and two studies reported an increase (Cao et al., 2015, 2017). An additional study examined the effect of DBS on cortico-subthalamic coherence (Oswal et al., 2016) and reported a decrease in this parameter. Notably, however, in all but one study (Cao et al., 2017) these effects were not found to be correlated with clinical improvement although all of the studies looked for such correlations. Cao et al. (2017) did find significant correlations between beta modulation by DBS and improvement of akinesia + rigidity but only for the right temporal channels. Without source localization it is difficult to say whether these effects are related to motor cortex beta. Thus the results of the majority of the studies suggest that beta decrease caused by DBS could be a side-effect of antidromic propagation of action potentials via the hyperdirect pathway rather than part of the mechanism causally related to clinical improvement. One possible interpretation is that it is the subcortical rather than cortical modulation that is key to achieving clinical improvement in PD. If the increase of cortical beta we report in the present study is causally related to clinical improvement, it is possible that DBS can bypass this mechanism and act on the downstream targets directly while disrupting cortical beta as a side effect. Alternatively, it could be that DBS and L-dopa have distinct mechanisms of action which affect cortical beta in the opposite ways. In either case, one would have to admit that generally there is no one-to-one relationship between cortical beta power and clinical state.

Our results, therefore, support the idea that cortical beta oscillations in humans are distinct from subcortical beta in the basal ganglia which has been consistently shown to increase in PD and undergo suppression by both L-dopa and DBS (Little and Brown, 2014). Furthermore, beta power in the STN was found to positively correlate with akinesia and rigidity in the OFF state (West et al., 2016) which is the opposite of what we found for cortical power. Simultaneous recordings from the cortex and the STN support this distinction. Although coherence was found between cortical motor areas and the STN in the beta band, the frequency of this coherence was higher than that of pathological beta and it was not decreased by L-dopa (Litvak et al., 2011a). The fact that in our study the increase in oscillatory power extended to 30 Hz (Fig. 3B) is also suggestive of a difference with subcortical beta where the effects of L-dopa were mainly manifest in the low beta band (13–22 Hz) (West et al., 2016). A study by a different group (Hirschmann et al., 2013) did report a decrease in M1-STN coherence with L-dopa, so the relation between cortical and subcortical beta oscillations in PD remains unclear. Hirschmann et al. also found a negative correlation between akinesia + rigidity scores and STN-cortex beta coherence, specific to the OFF state. This is very much in line with what was found in this study for cortical beta power and might speak for a reduction of cortical drive in the beta band as the disease progresses.

The noise levels in some of our MEG sensors differed between the recording blocks, making it necessary to correct the spectra at both the sensor and source level and this is a potential limitation of the study. In an event-related study a baseline could be used to correct for these effects but with resting-state recordings this was not a possibility. However, the FOOOF algorithm we used for isolating the oscillatory components of the spectra is a state-of-the-art approach for dealing with this confound. Using the high frequency part of the spectra for alignment across conditions is a crude approximation to the FOOOF approach which is computationally more feasible when dealing with beamformer images. We believe this is a valid approach since high gamma activity seen in MEG is not normally observed at rest (Crone et al., 2011) so the power above 100 Hz is a good estimate of the sensor noise.

Our study provides new evidence suggesting that the cortical beta oscillations should not be viewed as anti-kinetic. Their relation to clinical impairment in PD and the way they are affected by L-dopa is distinct from that of subcortical beta oscillations most commonly recorded in the STN. Comparison with previous studies suggests that the pattern of effects of PD and L-dopa on motor cortical beta may reverse over the course of the disease. A longitudinal study in the same patients recorded at different stages of PD would be necessary to confirm that this is the case and allow for the pinpointing of the disease stage at which this reversal may happen.

## Author contributions

Chunyan Cao was responsible for collection of MEG data. Chencheng Zhang obtained clinical video recordings. Bomin Sun, Shikun Zhan and Dianyou Li enrolled the primary PD patients and evaluated the UPDRS III scores. Vladimir Litvak wrote the script for the data analysis. Chunyan Cao and Vladimir Litvak analyzed the data and wrote the manuscript.

## Declaration of Competing Interest

The authors declare no potential conflict of interest.
